# Supplementary material for: SHAP-enhanced machine learning identifies modifiable obesity predictors across adolescent weight groups: A 2021 YRBSS analysis
Source: PLoS One. 2025 Oct 10;20(10):e0334502. doi: 10.1371/journal.pone.0334502 (PMC12513593; doi:10.1371/journal.pone.0334502)
Supplement: Attachment — Submitted filename: Response Letter.docx [file pone.0334502.s003.docx]

Additional Editor Comments:

Manuscript Title:

Machine Learning Analysis of Obesity Factors Across Weight Groups: Findings from the 2021 Youth Risk Behavior Surveillance System

General Assessment:

This manuscript addresses a timely and relevant public health issue—adolescent obesity—by applying supervised machine learning models to a large national dataset (YRBSS 2021). The incorporation of SHAP values to interpret feature importance is a methodological strength, offering transparency and clinical relevance. However, the manuscript requires substantial revision to clarify methodological choices, strengthen the scientific communication of results, and improve reproducibility and interpretability.

1. Title and Introduction

**Concerns:**

• The title does not reflect the use of SHAP or the study's focus on modifiable predictors, which are core to its novelty.

• The study aim appears too late in the introduction.

• The YRBSS dataset is not introduced early or described sufficiently.

• There is no justification provided for stratifying by weight categories.

**Recommendations:**

• Revise the title to reflect key methodological features. Suggested title:

“SHAP-Enhanced Machine Learning Identifies Modifiable Obesity Predictors Across Adolescent Weight Groups: A 2021 YRBSS Analysis.”

**Response:**

Thank you for this helpful suggestion. We have revised the title to highlight SHAP and modifiable predictors as advised.

• State the study objective clearly and early.

**Response:**

Thank you. We have moved the study objective earlier in the introduction to make it clear from the outset.

• Briefly describe the YRBSS dataset (e.g., scope, periodicity, population coverage).

**Response:**

Thank you. We have added a concise description of the YRBSS dataset earlier in the introduction.

• Justify the weight-group stratification using appropriate references (e.g., CDC percentile cutoffs for BMI-for-age).

**Response:**

Thank you. We now note in the introduction that participants were stratified by weight categories, and in the Methods section we provide justification using CDC BMI-for-age percentile cutoffs (BMIPCT).

**2. Methods**

**Concerns:**

• Unclear terminology (e.g., “two-step say technique”) may confuse readers.

• The definition of obesity and BMI percentile cutoffs is missing.

• Critical aspects of the machine learning pipeline are underreported:

No mention of hyperparameter tuning

No treatment of class imbalance

No description of cross-validation

•SHAP implementation details are insufficient.

• Reproducibility elements (software, random seed, code repository) are lacking.

**Recommendations:**

• Correct unclear terms or provide definitions.

**Response:**

Thank you. We have corrected the unclear term to “two-step imputation technique” and provided a brief explanation of how missing values were handled.

• Specify how BMI categories were defined and cite relevant standards (e.g., CDC, WHO).

**Response:**

Thank you. We have specified the definition of obesity and BMI percentile cutoffs in the Methods, citing CDC standards.

• Expand the ML pipeline description to include:

Hyperparameter optimization approach

Class imbalance techniques (e.g., SMOTE, weighting)

Validation strategy (e.g., k-fold CV, train-test split)

**Response:**

Thank you. We have revised the Methods to clarify our ML pipeline, including the random seed, software used, and SHAP implementation. We also explicitly state that we did not use hyperparameter optimisation, class imbalance techniques, or cross-validation, in order to prioritise transparency and reproducibility.

• Clearly explain how SHAP values were computed and aggregated (e.g., mean absolute values).

**Response:**

Thank you. We have clarified our SHAP implementation, specifying that SHAP values were aggregated using mean absolute values across all samples, and visualised with beeswarm plots.

• For reproducibility, list the software used, indicate random seed values, and share a code repository if available.

**Response:**

Thank you. We have added reproducibility details by specifying software versions, the random seed (set.seed = 3554), and a statement about code availability.

**3. Results**

**Concerns:**

• The results section includes redundant reporting (e.g., odds ratios repeated in text and tables).

• Overreporting of non-significant findings (e.g., soda, sleep) may distract from meaningful results.

• Performance metrics sensitive to class imbalance (e.g., F1-score, PR AUC) are missing.

• SHAP plots and nomograms are insufficiently labeled.

**Recommendations:**

• Streamline the text to highlight key, statistically significant findings.

**Response:**

Thank you. We have streamlined the Results section by highlighting only the key, statistically significant findings and moving non-significant results and redundant details to the supplementary materials.

• Move non-significant results to supplementary material.

**Response:**

Thank you. We have moved non-significant results to the supplementary materials and streamlined the Results section to emphasise only the key, statistically significant findings.

• Report additional evaluation metrics (e.g., F1-score: “F1 scores were 0.41 for Random Forest and 0.39 for XGBoost”).

**Response:**

Thank you. We have added imbalance-sensitive evaluation metrics, including weighted F1-scores (0.277 for Random Forest; 0.292 for XGBoost) and mean one-vs-rest AUC (0.575 and 0.571). We also reported class-level F1-scores to highlight the effect of class imbalance.

• Improve figure labeling:

Label SHAP axes (e.g., “SHAP value (impact on log-odds of obesity)”)

Clarify nomogram elements with legends or captions.

**Response:**

Thank you. We revised the figure labels and captions. SHAP plots now include axis labels and colour explanations, and the nomogram caption clarifies how points and predicted values are derived.

• State that top features were selected based on mean absolute SHAP values.

**Response:**

Thank you. We have clarified in the Results section that the top features were selected based on mean absolute SHAP values.

**4. Discussion**

**Concerns:**

• The discussion contains redundant commentary on interpretability.

• Terminology is inconsistent or unclear (e.g., “butter level,” “fresh BMI,” “quiet activities”).

• Overgeneralizations are made, particularly regarding dietary predictors.

• The narrative flow is disjointed.

• The limitations section is underdeveloped.

**Recommendations:**

• Consolidate discussion on SHAP and interpretability.

**Response:**

Thank you. We have consolidated the discussion on interpretability and SHAP into a single paragraph, reducing redundancy while emphasising its role in highlighting key predictors.

• Use consistent, scientific terminology (e.g., “butter level” → “milk intake,” “fresh BMI” → “BMI status”).

**Response:**

Thank you. We have revised inconsistent terminology, replacing unclear terms with consistent scientific expressions.

• Clarify overgeneralized findings (e.g., specify milk type/frequency).

**Response:**

Thank you. We have revised the discussion of dietary predictors to avoid overgeneralization, and clarified that the association with milk depends on both type (whole vs. low-fat) and frequency of intake.

• Improve discussion structure using subheadings (e.g., MVPA, screen time, substance use).

**Response:**

Thank you. We have reorganised the discussion with subheadings (e.g., MVPA, screen time, dietary predictors, substance use) to improve structure and readability.

• Expand the limitations section:

Cross-sectional design (no causal inference)

Self-reported data bias

Lack of objective activity measures (e.g., wearables)

Absence of longitudinal validation

**Response:**

Thank you. We have expanded the limitations section to address the cross-sectional design, reliance on self-reported data, lack of objective measures, and absence of longitudinal validation.

**5. Statistical Analysis Clarity**

**Concern:**

The authors mention the use of accuracy, sensitivity, specificity, and AUC to evaluate model performance but do not detail how these metrics were calculated or validated.

**Recommendation:**

• Briefly describe whether performance metrics were calculated on a holdout set or through cross-validation.

**Response:**

Thank you. We have clarified that all performance metrics (accuracy, sensitivity, specificity, AUC) were calculated on the held-out testing set.

• Indicate how classification thresholds were set.

**Response:**

Thank you. We have clarified that classification thresholds were set according to the default algorithm behaviour, assigning each case to the class with the highest predicted probability.

• Discuss the relevance of class imbalance metrics if applicable.

**Response:**

Thank you. We have added an explanation that weighted F1-score and mean one-vs-rest AUC are particularly relevant under class imbalance, as they provide a more balanced evaluation of model performance.

6. Minor Issues

• Proofread for minor typographical and grammatical inconsistencies.

**Response:**

Thank you. We have carefully proofread the manuscript to correct minor typographical and grammatical inconsistencies, ensuring consistent and clear language throughout.

• Standardize variable names and phrasing throughout the manuscript.

**Response:**

Thank you. We have standardised variable names and phrasing throughout the manuscript to ensure consistency.

**Final Recommendation:**

Major Revision

The manuscript presents a valuable application of machine learning to a pressing health issue, but critical revisions are necessary to ensure scientific clarity, methodological transparency, and interpretability. I encourage the authors to address the above comments comprehensively.

Please use the space provided to explain your answers to the questions above. You may also include additional comments for the author, including concerns about dual publication, research ethics, or publication ethics. (Please upload your review as an attachment if it exceeds 20,000 characters)

Reviewer #1: Thank you for the opportunity to review your manuscript. This study analyzes a large dataset, which is a valuable strength and holds great potential to contribute to the field. I appreciate the effort that went into collecting and analyzing the data.

That said, there are a few areas where the manuscript could be improved to better communicate your findings and enhance the overall impact of the paper. The structure and writing would benefit from revisions to improve clarity and logical flow. In some sections, there are grammatical issues and information that seems less directly relevant to your central research questions. A careful edit could help streamline the narrative and strengthen the presentation.

Additionally, while the dataset is robust, the findings in their current form are somewhat limited in novelty. The discussion section, in particular, could be expanded to explore the implications of your results more deeply and to connect them more clearly with existing literature. It may also be helpful to consider additional contextual factors—such as socioeconomic status—that could be influencing variables like ethnic group or breakfast frequency, as these were not fully addressed in the current version.

Overall, I believe the manuscript has potential, but it would benefit from substantial revision before being suitable for publication. I encourage you to continue refining your work and hope these comments are helpful in guiding your next steps.
