# Supplementary material for: SHAP-enhanced machine learning identifies modifiable obesity predictors across adolescent weight groups: A 2021 YRBSS analysis
Source: PLoS One. 2025 Oct 10;20(10):e0334502. doi: 10.1371/journal.pone.0334502 (PMC12513593; doi:10.1371/journal.pone.0334502)
Supplement: Attachment — Submitted filename: 2025.09.26 PLOS ONE Response Letter.docx [file pone.0334502.s004.docx]

PLOS ONE

Journal Requirements:

If the reviewer comments include a recommendation to cite specific previously published works, please review and evaluate these publications to determine whether they are relevant and should be cited. There is no requirement to cite these works unless the editor has indicated otherwise.

Additional Editor Comments:

Summary of Manuscript:

The manuscript examines factors associated with overweight and obesity among adolescents using data from the YRBSS dataset. The study applies multivariable logistic regression to evaluate associations between demographic, behavioral, and lifestyle variables and overweight/obesity status. Additionally, the authors present a predictive machine learning analysis using Random Forest and XGBoost models.

Major Comments:

Regression Analysis:

The logistic regression analyses are clearly presented with appropriate tables (Tables 2 and 3).

**Response**: Thank you!

Demographic and behavioral predictors (age, sex, race, MVPA, diet, sleep, and substance use) are analyzed correctly, and results are generally interpretable.

**Response**: Thank you!

Machine Learning Component:

The manuscript presents Random Forest and XGBoost results for obesity prediction.

**Response**: Thank you!

However, the editorial assessment indicates no hyperparameter tuning, no class imbalance handling, and no cross-validation were performed.

**Response**: Thank you and we addressed this concern by performing grid search for hyperparameter tuning, applying SMOTE with class weights for imbalance handling, and using repeated 5-fold cross-validation to ensure robust evaluation. Please see:

Reported performance metrics (accuracy ~0.66–0.67, very low positive predictive value) are not robust, limiting reliability and generalizability.

**Response**: Thank you and we have added up some analysis in the limitation section:

*“Additionally, model performance was modest (accuracy ~0.66–0.67, low positive predictive value), reflecting reliance on self-reported behavioural data. Despite tuning and imbalance handling, predictive power remained limited; thus, the ML analyses were intended to highlight correlates rather than provide precise prediction.”*

The current ML analysis is insufficient to support predictive claims.

Clarity and Structure:

Introduction and discussion sections have improved in clarity and flow.

**Response**: Thank you!

Methodology reporting is transparent for the regression analyses, but the ML methodology requires major improvements or reframing.

**Response**: Thank you. We have revised the *Statistical Analysis* section to clarify the framing of the machine learning methodology.

Minor Comments:

Tables are well organized; ensure consistent formatting for all p-values and odds ratios.

**Response**: Thank you, changed!

References are comprehensive and up-to-date.

**Response**: Thank you.

**Required Actions for Authors:**

Authors must address the critical limitations in the ML component. Two options are available:

**Perform a full ML re-analysis:**

Apply hyperparameter tuning (grid/random search).

Implement class imbalance strategies (SMOTE, class weighting).

Use robust validation (k-fold or nested cross-validation).

Update results, figures, and conclusions accordingly.

**Reframe the manuscript as descriptive/exploratory analysis:**

Focus on associations identified in regression analyses.

Remove or tone down all predictive claims.

Clearly state the absence of ML validation and tuning as a major limitation.

Once the manuscript is revised accordingly, it can be re-evaluated for potential acceptance.

**Response**: We appreciate this constructive suggestion. In line with the first option, we have conducted a full re-analysis of the ML component, including hyperparameter tuning (grid search with cross-validation), class imbalance handling (SMOTE with class weighting), and repeated 5-fold cross-validation. Results, figures, and conclusions have been updated accordingly, while the ML analyses are framed as complementary to regression findings.
